# Pharmacodynamic Impact of Carboxylesterase 1 Gene Variants in Patients with Congestive Heart Failure Treated with Angiotensin-Converting Enzyme Inhibitors

**DOI:** 10.1371/journal.pone.0163341

**Published:** 2016-09-23

**Authors:** Karl Emil Nelveg-Kristensen, Peter Bie, Laura Ferrero, Ditte Bjerre, Niels E. Bruun, Martin Egfjord, Henrik B. Rasmussen, Peter R. Hansen, INDICES Consortium

**Affiliations:** 1 Department of Cardiology, Gentofte University Hospital, Gentofte, Denmark; 2 Institute of Molecular Medicine, University of Southern Denmark, Odense, Denmark; 3 Institute of Biological Psychiatry, Mental Health Centre Sct. Hans, Copenhagen University Hospital, Roskilde, Denmark; 4 Department of Nephrology, Rigshospitalet, Copenhagen University Hospital, Copenhagen, Denmark; 5 Clinical Institute, Aalborg University, Aalborg, Denmark; University of Tampere, FINLAND

## Abstract

**Background:**

Variation in the carboxylesterase 1 gene (CES1) may contribute to the efficacy of ACEIs. Accordingly, we examined the impact of CES1 variants on plasma angiotensin II (ATII)/angiotensin I (ATI) ratio in patients with congestive heart failure (CHF) that underwent ACEI dose titrations. Five of these variants have previously been associated with drug response or increased CES1 expression, i.e., CES1 copy number variation, the variant of the duplicated CES1 gene with high transcriptional activity, rs71647871, rs2244613, and rs3815583. Additionally, nine variants, representatives of CES1Var, and three other CES1 variants were examined.

**Methods:**

Patients with CHF, and clinical indication for ACEIs were categorized according to their CES1 genotype. Differences in mean plasma ATII/ATI ratios between genotype groups after ACEI dose titration, expressed as the least square mean (LSM) with 95% confidence intervals (CIs), were assessed by analysis of variance.

**Results:**

A total of 200 patients were recruited and 127 patients (63.5%) completed the study. The mean duration of the CHF drug dose titration was 6.2 (SD 3.6) months. After ACEI dose titration, there was no difference in mean plasma ATII/ATI ratios between subjects with the investigated CES1 variants, and only one previously unexplored variation (rs2302722) qualified for further assessment. In the fully adjusted analysis of effects of rs2302722 on plasma ATII/ATI ratios, the difference in mean ATII/ATI ratio between the GG genotype and the minor allele carriers (GT and TT) was not significant, with a relative difference in LSMs of 0.67 (95% CI 0.43–1.07; P = 0.10). Results of analyses that only included enalapril-treated patients remained non-significant after Bonferroni correction for multiple parallel comparisons (difference in LSM 0.60 [95% CI 0.37–0.98], P = 0.045).

**Conclusion:**

These findings indicate that the included single variants of CES1 do not significantly influence plasma ATII/ATI ratios in CHF patients treated with ACEIs and are unlikely to be primary determinants of ACEI efficacy.

## Introduction

Activation of the renin-angiotensin-aldosterone system (RAAS) plays a pivotal role in cardiovascular disease and treatment with angiotensin-converting enzyme inhibitors (ACEIs), which inhibit the hydrolytic conversion of angiotensin I (ATI) to angiotensin II (ATII), forms an important part of the treatment for congestive heart failure (CHF), hypertension, and ischemic heart disease. ACEI treatment, however, is associated with substantial variability in efficacy, which cannot solely be explained by individual differences in clinical characteristics [1–8]. Although genetic diversity may contribute to such variability there is as yet very limited evidence available on this clinically important subject [9].

Most ACEIs are ester prodrugs, which are hydrolyzed to their active metabolites by hepatic carboxylesterase 1 (CES1) [10–12]. The activity of CES1 has been associated with marked individual variability and variants in the CES1 gene (*CES1*) as well as pharmacological CES1 inhibition have been shown to influence the CES1 hydrolytic capacity, and hence the activation of ACEIs [13–18]. The structure of *CES1* is complex. For example, *CES1* is subjected to duplication. The duplicated version of *CES1* is designated *CES1A2*, while *CES1A1* is the original gene copy [19]. Duplication of *CES1* has been associated with the pharmacokinetics of irinotecan in a dosage-dependent manner [20]. The haplotype of *CES1A2* with the ‘active promoter’, which is characterized by having two Sp1 transcription factor binding sites, has been associated with a higher transcriptional level of *CES1* that may lead to increased CES1 activity [18, 19]. On the other hand, a well-established non-synonymous missense single nucleotide polymorphism (SNP), rs71647871 (Gly143Glu), in *CES1A1* has been associated with decreased CES1 activity and reduced bioactivation of trandolapril [13]. In addition to ACEIs, CES1 is also important to the metabolism of clopidogrel, the anticoagulant prodrug dabigatran exitelate, and the central acting psychostimulant methylphenidate [21–23]. In this regard, rs2244613, which is located in a *CES1A1* intronic region, has been associated with decreased bioavailability of dabigatran, the activated metabolite of dabigatran exitelate, and reduced bleeding in dabigatran etxitelate-treated patients, and rs3815583 in the *CES1A1* promoter, has been linked to appetite reduction among ADHD patients treated with methylphenidate e [24, 25]. *CES1A1* also harbors a set of SNPs in its upstream part that are in strong LD with each other, including a SNP with a potential effect on the amount of enzyme produced, due to its localization in the Kozak sequence of the gene. To our knowledge, there are no reports available on the relationship between *CES1* variants and pharmacodynamic effects of ACEIs and it is notable that the plasma ATII/ATI ratio is closely correlated to circulating levels of active ACEI metabolites [26–29]. In this study we therefore examined the influence of the above-mentioned genetic variations in *CES1* on the plasma ATII/ATI ratio in ACEI-treated patients with CHF including nine of the SNPs in the upstream part of *CES1A1*, which form the so-called *CES1Var*. In addition, three selected *CES1* variants that were not suspected to have a functional impact themselves were included as potential markers of causal genetic variants.

## Materials and Methods

### Subjects

In the period 2012–2014 patients ≥18 years of age with CHF of any cause and a left ventricular ejection fraction ≤45% who were referred to the CHF outpatient clinic at Gentofte University Hospital, Copenhagen, Denmark, for initiation or dose titration of ACEIs and other CHF drugs were recruited for the study. Patients were routinely followed by outpatient consultations every second to fourth week until completion of CHF drug dose titration. Main exclusion criteria were treatment with captopril or lisinopril that are not metabolized by CES1 [10], treatment with angiotensin II type I receptor blockers, pregnancy, malignant disease, CHF requiring hospitalization, and baseline serum creatinine ≥150 mmol/l. At baseline blood samples (10 ml) were collected in EDTA-containing tubes for *CES1* genotyping. Patients were followed until they had been titrated to maximal tolerable doses of ACEIs, beta-blockers, and aldosterone antagonists, for at least two weeks or a maximum of 2 months [30]. On the day of study termination, blood samples (10 ml) were collected for determination of plasma ATI and ATII levels, and the plasma ATII/ATI ratio was calculated. Blood samples were collected during day time (9:00 AM–2:00 PM) in pre-chilled tubes containing EDTA and aprotinine, immediately centrifuged, and plasma stored at -20°C until analysis. The patients rested for 15–20 minutes in a sitting position prior to blood sample collection and the time of the last preceding ACEI drug ingestion was carefully recorded. Patients who stopped ACEI treatment during the follow up period were excluded and the causes for ACEI discontinuation, e.g., adverse events or non-compliance, were registered.

### Plasma angiotensin analyses

The plasma concentrations of ATI and ATII were determined by radioimmunoassay as described previously [31, 32]. Specific anti-ATI and anti-ATII antibodies, i.e., Ab-3-20008939 and Ab-5–030682 raised in rabbits were used in final dilutions of at least 1:100.000. There was <0.1% cross reactivity between these antibodies for ATI and ATII, but Ab-5–030682 cross-reacts with shorter bioactive angiotensins [31]. In brief, plasma samples were acidified by 4% acetic acid, extracted by use of C-18 Sep-Pak cartridges (Waters, Hedehusene, Denmark) and dried overnight. After elution, samples and antibodies were incubated for 24 hours, and known amounts of ^125^Iodine (I)-labeled ATI and ATII were then added for another 24 hours of incubation. Sediments of free antigen were obtained by adding a charcoal-plasma suspension followed by centrifugation. Finally, the radioactivity of the supernatant representing ^125^I bound antibodies was measured, the ATI and ATII concentrations were determined, and plasma ATI/ATII ratio was calculated.

### Genetic analyses

Genomic DNA was extracted from the EDTA-stabilized blood samples using the Maxwell^®^strument (Promega Corporation, Madison, WI, USA). Subsequently, we determined the total number of copies of *CES1A1* and *CES1A2* using a commercially available assay based on duplex real-time PCR (Thermo Fisher Scientific, Waltham, MA USA). This assay targeted intron 11 (TaqMan^®^ copy number assay Hs00139541_cn) in *CES1A1* and *CES1A2*. Since this region is identical in *CES1A1* and *CES1A2* the assay determined the number of copies of both of these gene versions. Deletion of *CES1A1* has not been reported. Hence, all individuals in our study were assumed to harbor two *CES1A1* copies. With this assumption, a copy number of three or four as determined by the above assay signified presence of one or two *CES1A2* copies.

For samples lacking *CES1A2* two overlapping long range PCRs were carried out allowing for the amplification of all *CES1A1* exons. The first of these long PCRs amplified a 12.5 kb fragment of the gene containing its promoter and exon 1–5. The second long PCR amplified a 19.2 kb *CES1A1* fragment containing exon 6–14. For samples with three or four copies, i.e. samples harboring *CES1A1* as well as *CES1A2*, the 12.5 kb fragment of *CES1A1* as well as the corresponding *CES1A2* fragment with the approximately same size was amplified. The long PCR for amplification of the *CES1A1* fragment containing exon 6–14 is unable to distinguish *CES1A1* from *CES1A2* and was therefore not applicable for analysis of samples containing both of these gene versions. The sequences of the forward and reverse primers for amplification of the 12.5 kb fragment of *CES1A1* were 5’-ACTATGGGGGGACGGAGTTCA-3’ and 5’-CCAGTCCTGAATTCAGGTATTGTAATCA-3’. The 12.5 kb fragment of *CES1A2* was amplified using the same reverse primer and a forward primer with the sequence 5’- CAGGAGCTATTGAGAGATGGAATCAT-3’. For amplification of the 19.2 kb fragment of *CES1A1* we used a forward and reverse primer with the sequences 5’-CTGATTACAATACCTGAATTCAGGAC-3’ and 5’-GTATTTCTGCTCATTATGGTCACG-3’, respectively. The amplified fragments were subjected to Sanger sequencing in order to determine the genotypes of the following *CES1A* SNPs: rs3815583, rs12149373, rs12149371, rs12149322, rs12149370, rs12149368, rs111604615, rs566557773, rs201577108, rs12149366, rs56278207, rs71647871, rs2302722, rs2244614, and rs2244613. We also identified the *CES1A2* variant with increased transcriptional activity, i.e., the haplotype carrying the ‘active promoter’ [18]. Our procedure was validated using a variety of different approaches. Notably, we showed that the *CES1A2* specific primers did not support amplification of samples that did not harbor this gene variant as determined by the real time PCR described above, and traces of *CES1P1* (the *CES1*-related pseudogene) or other undesired sequences were not detected in the Sanger chromatograms (not shown).

The single nucleotide variations rs12149368, rs12149373, rs12149371, rs12149322, rs12149370, rs111604615, rs566557773, rs201577108, and rs12149366, which all are located in the 5’ UTR or exon 1 of *CES1A1*, constitute a major haploblock. The variation rs12149368 resides at a position immediately upstream to the initiation codon of *CES1A1*, thereby altering the Kozak sequence in this gene. The variations rs111604615, rs201577108 and rs12149366 all cause an amino acid change. Consequently, we hypothesized that the abovementioned major haploblock was associated with altered CES1 activity [33]. The single nucleotide variation rs56278207, which is located in intron 1 of *CES1A1*, is in LD with the variations in the major haploblock and served as an additional marker of this block. Similarly, rs2244614 of *CES1A1*, which is in linkage disequlibrium with rs2244613, was included in the analysis. Finally, intronic rs2302722 was selected as another marker since its genotype proportion was compatible with those expected under Hardy-Weinberg equilibrium (HWE) and the minor allele frequency (MAF) of this single nucleotide variation was found to exceed 0.05.

Alignment of sequences and detection of genetic variants were done using Lasergene (DNASTAR, Madison, WI, USA). For mapping we applied Geneious v7.1.5 (Biomatters, Auckland, New Zealand). *CES1A1* sequences were aligned to Hg19 (GRh37.p13) and *CES1A2* sequences were aligned with AB119998.1. The *CES1A2* variant with the “active promoter” was identified based upon previously published sequence information [18]. Measures of pairwise linkage disequilibrium (LD) expressed as *r*^*2*^ were calculated and visualized using Haploview v4.2 [34]. The LD blocks were identified using the confidence interval method implemented in Haploview. The Chi-square test was used to examine whether genotype proportions corresponded to those expected under Hardy-Weinberg equilibrium.

### Statistics

One-way analyses of variance (ANOVA) were applied to model the differences between genotype groups in means for the plasma ATII/ATI ratio. Results from the ANOVA models values were given as least square means (LSMs). To fit the assumption of a normally distributed outcome variable, values of the plasma ATII/ATI ratio were transformed into a base 10 logarithmic scale before statistical assessment. Initially, all genetic variants were assessed in univariate analyses. Only variants that produced a univariate significance level of P≤0.30 were further investigated in multivariate ANOVA models adjusted for age, sex, height, blood pressure, treatment with spironolactone or eplerenone, treatment with beta-blockers and, ultimately, also adjusted for the timespan from last ACEI tablet ingestion until blood sample collection at the day of study termination. The single nucleotide variants under examination were bi-allelic and thus giving rise to three genotypes each. Hence, these variants were initially included as three-level variables. By contrast, the *CES1A2* haplotype with the ‘active promoter’ was exclusively examined as a binary (active vs. inactive) variable [18]. In the adjusted models, the genetic variants with three genotypes were condensed into dichotomous variables, i.e., carriers or non-carriers of the minor allele, to increase the statistical power of the study. Sensitivity analyses were done in a sub-group of enalapril-treated patients. The absence of interaction between variables and the assumption of exposure group equality of the standard deviation were fulfilled if not otherwise stated. Due to multiple testing, we used a Bonferroni-corrected significance level of P≤0.01 (standard P-value of 0.05 divided by the number of independent analyses) for evaluation of the association between *CES1* variants and the outcome measure. SNPs located in a haploblock are not independent and for such SNPs we therefore only corrected once. Variants with low minor allele frequencies (MAFs) were excluded from the association analyses. We used a Bonferroni-corrected significance level of P≤0.003 for the assessment of P values in the Hardy Weinberg equilibrium (HWE) tests since these included all 15 examined *CES1* SNP genotypes and the copy number variation. Analyses and data management were performed in SAS version 9.3 (SAS Institute Inc. Cary, North Carolina).

### Ethics

This study was approved by the Danish National Committee on Health Research Ethics (Protocol no.: H-4-2012-094) and the Danish data protection agency (I-suite no.: 01825 and identification no.: GEH-2012-032), respectively. All participants gave their written informed consent before inclusion.

## Results

### Study population and CES1 genetics

**Fig 1:**
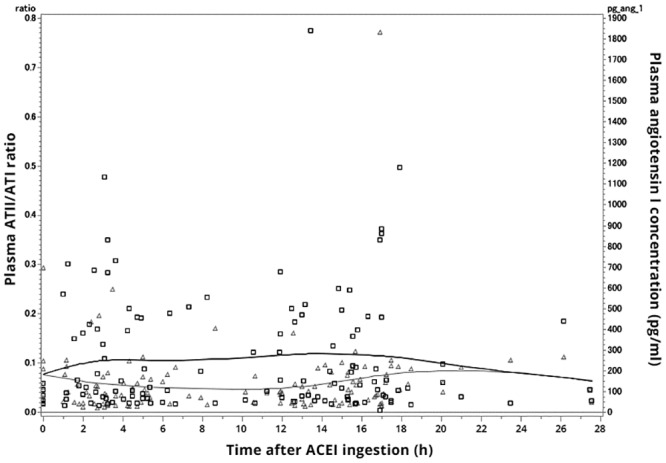
Plasma angiotensin I (ATI) concentration and plasma ATII/ATI ratio after angiotensin-converting enzyme inhibitor (ACEI) ingestion in patients with congestive heart failure after completion of dose titration. Values are plotted against the time in hours from ACEI (enalapril, ramipril, or trandolapril) ingestion until blood sample collection. Δ: Plasma ATII/ATI ratio, □: Plasma ATI concentrations.

**Fig 2:**
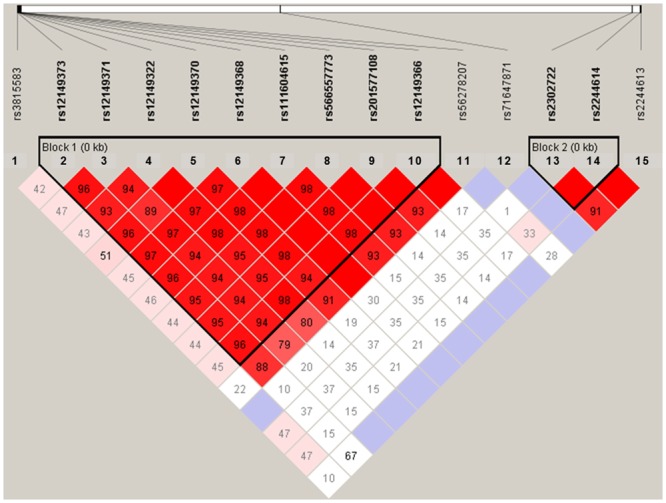
Linkage disequilibrium (LD) relationships between single nucleotide polymorphisms (SNPs) of *CES1A1*. The top of the figure shows SNPs and the bottom of the figure shows LD relationships as well as LD blocks of highly coupled variants. The strength of LD was determined by R^2^ statistics.

A total of 200 patients with CHF were recruited for the study. Hereof, 50 (25%) patients were excluded because they discontinued their ACEI treatment before the final CHF drug dose titration was achieved, 9 (4.5%) failed to show up for scheduled appointments, 8 (4.0%) were excluded due to severe comorbidity (liver cirrhosis [n=1] and cancer [n=4]) or potential ACEI adverse effects (kidney failure [n=1], hyperkalemia [n=1], and symptomatic hypotension [n=1]), and 6 (3.0%) patients died, leaving 127 (63.5%) subjects available for analysis. Of these 127 patients, 99 (78%) were treated with enalapril, 24 (19%) with ramipril, and 4 (3%) with trandolapril, respectively. The mean duration of the CHF drug dose titration period was 6.2 (SD 3.6) months. Other baseline characteristics of subjects in the total and analyzed study population are shown in Table 1. As shown in Fig 1, there was a time-dependent and inverse relationship between plasma ATI levels and the plasma ATII/ATI ratio (P for interaction = 0.026).The distribution of *CES1A2* with 2 (n = 89), 3 (n = 34), and 4 (n = 4) *CES1* copies, respectively, were in HWE (P = 0.73). Allele frequencies of the *CES1A1* variants are shown in Table 2. Plasma levels of ATI and ATII, and the plasma ATII/ATI ratio, respectively, in subjects with each of the *CES1* variants are shown in Table 3. The single nucleotide variants at rs12149373, rs12149322, rs111604615, rs566557773, rs201577108, rs12149366 were not in HWE (P<0.003) and the frequencies of the minor allele at rs71647871 and the “active promoter” of *CES1A2* were low (0.01 [n=2] and 0.03 [n=2], respectively) (Table 2). Accordingly, these three variants were not considered for further statistical analysis. Similarly, data on the *CES1A2* ‘active promoter’ was not included in Table 2 as this haplotype was rare in our study population. The reference alleles of rs56278207, rs2244613 and rs2244614 were not the major alleles [35]. The majority of the investigated SNPs within *CES1A1* were highly correlated as evidenced by high R^2^ values in the pairwise comparisons as shown in Fig 2. Data were missing for 41 individuals in the analyses of rs2302722, rs2244614, and rs2244613, respectively, which reflected that the long PCR for amplification of exon 6–14 of *CES1A1* is unable to discriminate *CES1A1* from *CES1A2* and therefore was not applicable for analysis of *CES1A1* in individuals carrying *CES1A2* (Table 2).

**Table 1:** Baseline characteristics of the analyzed study population (n = 127).

Female (n, [%])	30 (23.0)
Age (mean [SD] years)	68.50 (11.7)
Systolic BP (mmHg, mean [SD])	125.31 (17.9)
Diastolic BP (mmHg, mean [SD])	73.12 (11.3)
Height (cm, mean [SD])	176.48 (9.0)
BMI (kg/m^2^, mean [SD])	26.29 (4.6)
NYHA class (mean [SD])	1.46 (0.6)
EF (mean [SD])	0.38 (0.11)
PCI (n, %)	38 (29.9)
CABG (n, %)	21 (16.5)
DM type 1 (n, %)	2 (1.5)
DM type 2 (n, %)	20 (15.7)
Atrial fibrillation (n, %)	54 (42.5)
IHD (n, %)	66 (52.0)
COPD (n, %)	20 (15.7)
History of moking (n, %)	75 (59.1)
Enalapril (n, %)	99 (78.0)
Ramipril (n, %)	24 (18.9)
Trandolapril (n, %)	4 (3.1)
Beta-blockers (n, %)	110 (86.6)
Aldosterone inhibitors (n, %)	43 (38.9)

BMI: body mass index; BP: blood pressure; CABG: coronary artery bypass graft surgery; COPD: chronic obstructive pulmonary disease; DM: diabetes mellitus;

EF: Left ventricular ejection fraction; IHD: ischemic heart disease; NYHA: New York Heart Association; PCI: percutaneous coronary intervention; SD: standard deviation.

**Table 2:** *CES1A1* variants: allele and genotype frequencies for the analyzed study population (n = 127).

SNP	Position	Reference allele	Minor allele	Major allele	MAF	Missing (n)	Genotype (n/n/n)	P-value (HWE)
rs3815583	Promoter	T	G	T	0.20	7	8/32/80	0.068
rs12149373	Promoter	A	G	A	0.23	7	15/24/81	0.000
rs12149371	Promoter	A	G	A	0.19	7	9/28/83	0.007
rs12149322	Promoter	G	C	G	0.21	7	11/28/81	0.001
rs12149370	Promoter	A	G	A	0.18	7	5/34/83	0.556
rs12149368	Promoter	C	G	C	0.14	7	6/22/92	0.007
rs111604615	Exon	G	C	G	0.20	7	11/27/82	0.001
Rs566557773	Exon	C	T	C	0.20	7	11/27/82	0.001
rs201577108	Exon	T	C	T	0.20	7	11/27/82	0.001
rs12149366	Exon	T	G	T	0.20	7	10/27/83	0.002
rs56278207	Intron	-	-	T	0.33	9	18/42/58	0.034
rs71647871	Exon	G	A	G	0.01	4	0/2/121	0.927
rs2302722	Intron	G	T	G	0.10	41	2/13/71	0.160
rs2244614	Intron	C	C	T	0.31	41	8/37/41	0.933
rs2244613	Intron	C	C	A	0.04	41	0/7/79	0.694

SNP: single nucleotide polymorphism; MAF: minor allele frequency; HWE: Hardy-Weinberg equilibrium.

**Table 3:** Plasma angiotensin I (ATI) and ATII concentrations, and plasma ATII/ATI ratio.

Gene variation	Genotype	AT I (pg/ml; mean[SD])	AT II (pg/ml; mean[SD])	AT II/I-ratio (mean[SD])	P- Value^a^	P-Value^b^
CES1A2 promoter					-	-
	Weak	234.7 (31.2)	8.3 (8.6)	0.072 (0.134)		
	Strong^c^	59.1 (1.4)	4.2 (2)	0.071 (0.035)		
		-	-	-		
Copy number					0.47	0.22
	2	257.1 (258.3)	8.5 (8.3)	0.056 (0.049)		
	3	242.8 (354.6)	8.3 (6.9)	0.081 (0.133)		
	4	296.3 (383.7)	8.1 (2.4)	0.065 (0.044)		
rs3815583 (T>G)					0.81	0.62
	TT	233.0 (244.8)	8.3 (7.6)	0.067 (0.093)		
	TG	266.2 (353.5)	8.5 (9.2)	0.057 (0.044)		
	GG	290.6 (386.9)	7.7 (3.2)	0.066 (0.058)		
rs71647871 (G>A)					-	-
	GG	253.6 (289.2)	8.5 (7.9)	0.063 (0.08)		
	GA	171.4 (169.1)	8.5 (5.2)	0.068 (0.036)		
	AA	-	-	-		
rs2244613 (C>A)					0.42	-
	AA	267.4 (66.5)	8.6 (8.5)	0.056 (0.051)		
	CA	119.9 (112.3)	6.6 (7.3)	0.054 (0.015)		
	CC	-	-	-		
rs2244614 (C>T)					0.64	0.39
	TT	210.3 (188.4)	8.1 (7.7)	0.056 (0.055)		
	CT	261.9 (300.6)	8.6 (9.6)	0.056(0.047)		
	CC	258.3 (236.4)	9.3 (6.1)	0.06 (0.031)		
rs12149373 (A>G)					0.09	0.23
	AA	225.6 (242.2)	8 (7.5)	0.066 (0.091)		
	AG	329.7 (386.2)	9.5 (10.3)	0.044 (0.031)		
	GG	219.6 (311.8)	7.9 (3.8)	0.085 (0.07)		
rs12149371 (A>G)					0.75	0.58
	AA	224.8 (242.8)	8 (7.4)	0.066 (0.09)		
	AG	284.1 (365.4)	9.3 (9.7)	0.06 (0.054)		
	GG	225.6 (242.2)	7.7 (3.9)	0.059 (0.059)		
rs12149322 (G>C)					0.49	0.63
	GG	211.6 (227.3)	8 (7.5)	0.066 (0.091)		
	GC	317.1 (399.0)	9.5 (9.7)	0.052 (0.039)		
	CC	225.6 (242.2)	7.5 (3.6)	0.079 (0.077)		
rs12149370 (A>G)					0.69	0.65
	AA	225.6 (295.3)	8 (7.5)	0.066 (0.091)		
	AG	304.2 (377.9)	9.2 (8.9)	0.058 (0.051)		
	GG	172.5 (169.8)	6.9 (2.9)	0.073 (0.07)		
rs12149368 (C>G)					0.80	0.88
	CC	247.5 (295.3)	8.2 (7.5)	0.063 (0.086)		
	CG	214.4 (196.2)	9 (9.8)	0.07 (0.058)		
	GG	332.9 (421.3)	8 (3.7)	0.063 (0.068)		
rs111604615 (G>C)					0.77	0.65
	GG	233.4 (250.8)	8.1 (7.5)	0.065 (0.091)		
	GC	273.1 (356.8)	9.3 (9.8)	0.054 (0.038)		
	CC	269.9 (353.9)	7.8 (3.9)	0.079 (0.078)		
rs566557773 (C>T)					0.90	0.65
	CC	233.4 (250.8)	8.1 (7.5)	0.065 (0.091)		
	CT	259.7 (356.4)	9.4 (9.8)	0.063 (0.053)		
	TT	310.3 (351.6)	7.5 (3.7)	0.058 (0.055)		
rs201577108 (T>C)					0.90	0.86
	TT	233.4 (250.8)	8.1 (7.5)	0.065 (0.091)		
	TC	256.7 (356.4)	9.4 (9.8)	0.063 (0.053)		
	CC	310.3 (351.6)	7.5 (3.7)	0.058 (0.055)		
rs12149366 (T>G)					0.67	0.93
	TT	237.7 (252.3)	8.1 (7.4)	0.065 (0.09)		
	TG	236.5 (352.2)	9.2 (9.9)	0.065 (0.052)		
	GG	336.7 (358.9)	7.9 (3.7)	0.055 (0.057)		
rs56278207 (ins[T])					0.78	0.62
	TT	247.7 (311)	8.8 (7.9)	0.073 (0.105)		
	-T	244.2 (271.1)	8.6 (9)	0.06 (0.051)		
	—	210.3 (239.3)	6.1 (3.3)	0.05 (0.029)		
rs2302722 (C>T)					0.27	0.10
	GG	279.1 (274.2)	8.7 (8.9)	0.055 (0.052)		
	GT	122.5 (130.9)	5.5 (3)	0.065 (0.038)		
	TT	278.5 (125.9)	16.5 (9.2)	0.058 (0.007)		

SD: standard deviation.

^a^Genetic variants included as three leveled variables.

^b^Genetic variants included as two leveled variables.

^c^Active promoter

### Influence of CES1 gene variations on the plasma ATII/ATI ratio

**Fig 3:**
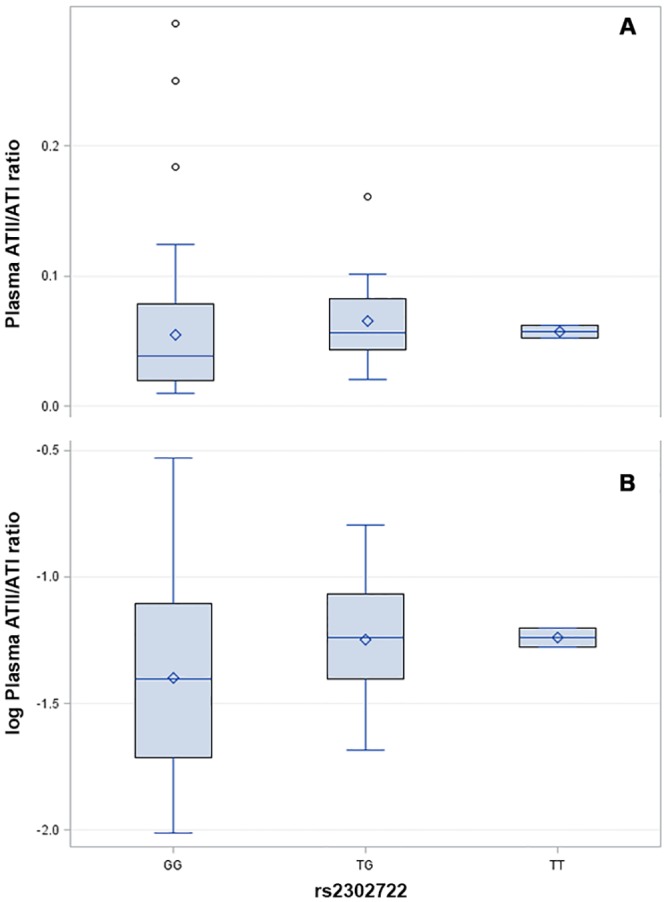
Distribution of the plasma angiotensin II (ATII)/ATI ratios (A) and logarithmic-transformed ATII/ATI ratios (B) according to *CES1A1* rs2302722 genotypes.

There were no statistical differences between the means of plasma ATII/ATI ratios from subjects with any of the investigated *CES1* variations when these were included either as three- or two-level variables, respectively. Only rs2302722 (P = 0.27) complied with the pre-specified limit (P <0.30) for further assessment in multivariate ANOVA models (Table 3). Mean plasma ATII/ATI ratios among subjects with each of the three genotypes at rs2302722 and the logarithmic-transformed values of the plasma ATII/ATI ratios used for the statistical analyses are provided in Fig 3. As only two subjects were homozygous for the minor allele (T) at rs2302722, the GT and TT genotypes were analyzed as a single group. In the fully adjusted analysis of the effect of the rs2302722 genotype, the mean plasma ATII/ATI ratios for the GG genotype (LSM 0.041 [95% confidence interval (CI) 0.031–0.055]) and the minor allele carriers, i.e., GT and TT (LSM 0.059 [95% CI 0.037–0.096]) did not significantly differ from each other, with a relative difference in LSMs of 0.67 (95% CI 0.43–1.07; P = 0.10). In analyses restricted to only include enalapril-treated patients that constituted the vast majority of our study population (7 8%), the relative difference between LSMs for the GG (0.039 [95% CI 0.028–0.054]) and GT (0.064 [95% CI 0.036–0.112]) remained non-significant after correction for multiple comparisons (0.60 [95% CI 0.37–0.98], P = 0.045). There were no enalapril-treated individuals who were homozygous for the minor allele (TT) of rs2302722. Hence, this genotype was not represented in the latter analysis.

## Discussion

In this study of patients with CHF that underwent dose titration with ACEIs that are activated by CES1, we investigated the impact of a total of 17 selected *CES1* variations on the plasma ATII/ATI ratio, a proximal pharmacodynamic marker of ACEI activity. Genetic analysis confirmed a high level of LD between several of the investigated *CES1* variations. Furthermore, we found no significant association between the examined genotypes and the plasma ATII/ATI ratio, when data were assessed in univariate unbalanced ANOVA models. In the subsequent multivariate analyses of the effect of rs2302722 (the only *CES1* variant that qualified for final analyses) and in models that exclusively included enalapril-treated patients, respectively, results remained non-significant based on the Bonferroni-corrected significance level for multiple comparisons. To our knowledge, we are the first to investigate the impact of *CES1* variation on a pharmacodynamic outcome parameter of ACEI treatment and although more studies are clearly warranted, the results suggest that the investigated variants in *CES1* are unlikely to be major determinants of ACEI efficacy.

Due to the pivotal role of CES1 in the hydrolytic activation of most ACEIs and an increased focus on individually tailored cardiovascular treatment, pharmacogenetic research on the importance of *CES1* variation for the response to ACEI treatment, as well as studies of CES1-mediated drug-drug interactions, has lately received increasing interest. Numerous variants within the entire gene encoding CES1 have been reported of which some have been shown to reduce or increase *CES1* transcription and CES1 activity [18, 19, 36]. However, as yet only very few *CES1* variants have been associated with altered pharmacokinetics of ACEIs and clinical outcomes among ACEI-treated patients. The non-conservative single nucleotide substitution variation at codon 143 in exon 4 of *CES1A1* that results in a change of the nucleotide G to an A (rs71647871) was associated with marked reduction of CES1 *in vitro* activity and complete inhibition of the hydrolytic conversion of trandolapril to the active metabolite trandolaprilat [13]. This variation has also been associated with reduced *in vitro* hydrolysis of other ACEI prodrugs, i.e., enalapril, ramipril, perindopril, moexipril, and fosinopril [36, 37]. To our knowledge, only the SNP -816 A>C at *CES1A2* has been associated with a clinical outcome measure in patients treated with ACEIs, i.e., a reduction of the antihypertensive effect of imidapril in a relatively small (n = 105) study of Japanese patients with hypertension [38]. However, a subsequent study of Japanese cancer patients revealed the SNP -816 A>C to reside in *CES1P1* and being absent or rare in *CES1A2*, thus questioning the exact nature of the association with the response to imidapril [20].

The HWE testing of several of the SNPs produced low P values which could reflect genotyping inaccuracies or non-random sampling of our study population. However, since the applied genotyping procedure has been extensively validated in our laboratory without giving rise to suspected co-amplification of undesired DNA fragments, e.g., fragments of *CES1P1* or mixed reads in the Sanger sequencing chromatograms (data not shown), it is highly unlikely that genotyping inaccuracies were primary determinants of these low P values. Instead, we focused our attention on the potential pathophysiological functions of CES1. Besides having a role in the hydrolytic conversion of various drugs, CES1 is involved in several endogenous physiological processes, e.g., hydrolytic conversion of cholesteryl esters and triacylglycerol,[39–43] fatty acyl coenzyme A hydrolysis,[44] and fatty acid ethyl ester synthase activity,[44, 45] and may therefore be implicated in development of cardiovascular disease, e.g. by contributing to dyslipidemia associated with increased risk of ischaemic heart disease.[46] Accordingly, the low P values observed upon HWE testing of several of the SNPs could reflect that patients with certain *CES1* genotypes and CHF disease subtypes were preferentially recruited in the study. Also, the scientific value of analyses of SNPs that are in strong LD may be limited as these to some extent represent redundant analyses. However, due to the observed minor differences in MAFs between the SNPs in the LD block, which are all solitary markers of the haplotype carrying *CES1VAR*, we found it reasonable to report the results of all the included genetic variations. In the present study, although the subanalysis of enalapril-treated patients produced a P value of 0.045, the results of the fully adjusted models of rs2302722 did not reach sufficient levels of significance, particularly not after Bonferroni correction for multiple parallel comparisons. Nonetheless, rs2302722 represented the most promising pharmacogenetic variant of our current enquiry.

In the current study, we did not find any association between *CES1* variants and the plasma ATII/ATI ratio when all ACEIs were included in the model, and the results did not change when genetic variants were condensed into dichotomous variables or in multivariate analyses where only enalapril-treated patients were included, respectively. These results are in line with a recently published *in vitro* study that found no association between *CES1* copy number variation and CES1 activity, and where the nonsynonymous *CES1* variants G19V, S83L and A270S had no influence on ACEI activation by CES1 [36]. Conversely, our results may be contrary to previously published results on a significant effect of rs2244613 on dabigatran activation and bleeding, and the association between rs3815583 and appetite reduction in patients treated with methylphenidate, respectively [24, 25]. However, the potential impact of these two genetic variations on *CES1* expression and CES1 hydrolytic activity towards ACEIs or other substrates is not yet known, and such observed effects might reflect LD with causal genetic variants related to the efficacy and safety of these drugs. Also, although the correlation between plasma ACE activity and the systemic (plasma) ATII/ATI ratio is well documented, systemic activation of the renin-angiotensin system does not necessarily reflect tissue specific ACE activity, which is also dependent on the disease etiology and other pathogenic mechanisms [28, 29, 47]. In this regard, a previous study has found that myocardial, but not systemic, pulmonary or renal ACE activities were increased in a rodent model of CHF, and human cardiac ACE gene expression has been shown to be increased among patients with CHF compared to persons with normal hearts [48, 49]. Intriguingly, there are also studies to suggest that tissue specific conversion of ATI to ATII increases over time despite treatment with ACEIs, and that intracellular ATII synthesis as well as the mediation of the more prolonged genomic effects of ATII, e.g., translation of growth factors and immunomodulatory cytokines, is independent of ACE activation and ATII receptor type 1 binding, respectively [50, 51]. Furthermore, an aldosterone escape has been reported in several studies with rising systemic levels of aldosterone during prolonged treatment with ACEIs in patients with CHF [52]. Accordingly, the negative findings of the present study do not allow for conclusions on effects of *CES1* variations on specific effects of ACEIs.

Although the therapeutic actions of ACEIs are considered to represent a drug class effect, the molecular structures of these drugs are distinct, which may affect their individual pharmacokinetic and pharmacodynamic properties [53, 54]. Also, the tissue penetration has been shown to vary between ACEIs as a consequence of their respective lipophilicity [55]. A previous *in vitro* study also suggested that the efficacy of CES1-mediated hydrolysis of enalapril was inferior to that of ramipril and trandolapril, and that these drugs exhibited different types of CES1 enzyme kinetics [14]. Likewise, another recent study of healthy volunteers showed a 20% reduction of enalaprilat (the activated form of enalapril) concentration in subjects homozygous for the minor allele at rs71647871, whereas no observable effect of this SNP was found on the pharmacokinetics of quinapril [37]. Clopidogrel is also a substrate for CES1, and after ingestion more than 90% of this prodrug is hydrolyzed to an inactive metabolite by hepatic CES1, thus escaping cytochrome P450-mediated activation [23]. Importantly, *in vitro* studies have shown that enalpril and trandolapril inhibited the CES1-mediated hydrolysis of clopidogrel to the deesterified and inactive metabolite, which was translated into an increased risk of clinically significant bleeding in patients with acute myocardial infarction co-treated with clopidogrel and ACEIs [17]. However, these results have subsequently been challenged and the sum of current evidence would appear to indicate that although *CES1* variation may account for some variability of CES1 enzymatic activity between individuals, the frequency and the effect size of the variants of *CES1* are likely to be small. Hence, these variants may have limited clinical relevance [56].

### Strengths and limitations

The present study was restricted by a relatively small sample size and hence had limited statistical power. Consequently, we were unable to do analyses stratified for individual ACEIs and ACEI doses, respectively. However, we found no significant differences in maintenance doses of enalapril (the most frequently used ACEI) between the investigated *CES1* variants (not shown). In addition, low P values were obtained by HWE testing of several SNPs which complicated the interpretation of some of the findings. In contrast to previous studies on plasma ATI and ATII levels in ACEI-treated patients, where subjects rested in a supine position before blood samples were collected, we applied a sitting position that may have influenced AT levels [57]. However, the plasma ATII/ATI ratio and the relationship between this ratio and circulating levels of ATI during ACEI treatment observed in the present study were comparable to previous findings [29, 58]. Also, environmental factors may have affected the impact of *CES1* variants and ACEI treatment on the plasma ATII/ATI ratio, including dietary habits, and results might have been influenced by CES1-dependent interactions with endogenous CES1 substrates and other CES1-metabolised drugs often used for patients with CHF, e.g., simvastatin and carvedilol [59–62]. Furthermore, patients with CHF frequently have altered drug pharmacokinetics owing to, e.g., intestinal congestion, reduced organ perfusion, and impaired renal and hepatic drug clearance, which also may have affected the results [63]. Also, as the current study did not examine all known genetic variants of *CES1*, we cannot exclude the possibility that undetermined *CES1* variants may have contributed to the variability in ACEI prodrug activation and subsequent pharmacodynamics.

## Conclusion

The present study of patients with CHF that underwent ACEI dose titration did not support an association between a range of *CES1* variants and ACEI pharmacodynamics measured by the plasma ATII/ATI ratio. These findings indicate that the investigated variants in *CES1* are unlikely to be primary determinants of ACEI efficacy.

## References

[1] Kober L, Torp-Pedersen C, Carlsen JE, Bagger H, Eliasen P, Lyngborg K, et al (1995) A clinical trial of the angiotensin-converting-enzyme inhibitor trandolapril in patients with left ventricular dysfunction after myocardial infarction. Trandolapril Cardiac Evaluation (TRACE) Study Group. N Engl J Med 333: 1670–6. 7477219 10.1056/NEJM199512213332503

[2] Webb AJ, Fischer U, Mehta Z, Rothwell PM. (2010) Effects of antihypertensive-drug class on interindividual variation in blood pressure and risk of stroke: a systematic review and meta-analysis. Lancet 375: 906–15. 10.1016/S0140-6736(10)60235-8 20226989

[3] Elliott WJ. (1996) Higher incidence of discontinuation of angiotensin converting enzyme inhibitors due to cough in black subjects. Clin Pharmacol Ther 60: 582–8. 8941032 10.1016/S0009-9236(96)90155-1

[4] Schelleman H, Stricker BH, De Boer A, Kroon AA, Verschuren MW, Van Duijn CM, et al (2004) Drug-gene interactions between genetic polymorphisms and antihypertensive therapy. Drugs 64: 1801–16. 15301563 10.2165/00003495-200464160-00006

[5] Materson BJ. (2007) Variability in response to antihypertensive drugs. Am J Med 120: S10–20. 10.1016/j.amjmed.2007.02.003 17403377

[6] Svanstrom H, Pasternak B, Melbye M, Hviid A. (2015) Use of different types of angiotensin converting enzyme inhibitors and mortality in systolic heart failure. Int J Cardiol 182: 90–6. 10.1016/j.ijcard.2014.12.092 25576729

[7] van Vark LC, Bertrand M, Akkerhuis KM, Brugts JJ, Fox K, Mourad JJ, et al (2012) Angiotensin-converting enzyme inhibitors reduce mortality in hypertension: a meta-analysis of randomized clinical trials of renin-angiotensin-aldosterone system inhibitors involving 158,998 patients. Eur Heart J 33: 2088–97. 10.1093/eurheartj/ehs075 22511654 PMC3418510

[8] Savarese G, Costanzo P, Cleland JG, Vassallo E, Ruggiero D, Rosano G, et al (2013) A meta-analysis reporting effects of angiotensin-converting enzyme inhibitors and angiotensin receptor blockers in patients without heart failure. J Am Coll Cardiol 61: 131–42. 23219304 10.1016/j.jacc.2012.10.011

[9] Talameh JA, Lanfear DE. (2012) Pharmacogenetics in chronic heart failure: new developments and current challenges. Curr Heart Fail Rep 9: 23–32. 10.1007/s11897-011-0076-2 22135185 PMC3917307

[10] DrugBank. Available: http://www.drugbank.ca/.

[11] Rasmussen HB, Bjerre D, Linnet K, Jurgens G, Dalhoff K, Stefansson H, et al (2015) Individualization of treatments with drugs metabolized by CES1: combining genetics and metabolomics. Pharmacogenomics 16: 649–65. 10.2217/pgs.15.7 25896426

[12] Merali Z, Ross S, Pare G. (2014) The pharmacogenetics of carboxylesterases: CES1 and CES2 genetic variants and their clinical effect. Drug Metabol Drug Interact 29: 143–51. 10.1515/dmdi-2014-0009 24988246

[13] Zhu HJ, Appel DI, Johnson JA, Chavin KD, Markowitz JS. (2009) Role of carboxylesterase 1 and impact of natural genetic variants on the hydrolysis of trandolapril. Biochem Pharmacol 77: 1266–72. 10.1016/j.bcp.2008.12.017 19185566

[14] Thomsen R, Rasmussen HB, Linnet K, Consortium I. (2014) In vitro drug metabolism by human carboxylesterase 1: focus on angiotensin-converting enzyme inhibitors. Drug Metab Dispos 42: 126–33. 10.1124/dmd.113.053512 24141856

[15] Takai S, Matsuda A, Usami Y, Adachi T, Sugiyama T, Katagiri Y, et al (1997) Hydrolytic profile for ester- or amide-linkage by carboxylesterases pI 5.3 and 4.5 from human liver. Biol Pharm Bull 20: 869–73. 9300133 10.1248/bpb.20.869

[16] Sato Y, Miyashita A, Iwatsubo T, Usui T. (2012) Simultaneous absolute protein quantification of carboxylesterases 1 and 2 in human liver tissue fractions using liquid chromatography-tandem mass spectrometry. Drug Metab Dispos 40: 1389–96. 10.1124/dmd.112.045054 22504157

[17] Kristensen KE, Zhu HJ, Wang X, Gislason GH, Torp-Pedersen C, Rasmussen HB, et al (2014) Clopidogrel bioactivation and risk of bleeding in patients cotreated with angiotensin-converting enzyme inhibitors after myocardial infarction: a proof-of-concept study. Clin Pharmacol Ther 96: 713–22. 10.1038/clpt.2014.183 25222620

[18] Yoshimura M, Kimura T, Ishii M, Ishii K, Matsuura T, Geshi E, et al (2008) Functional polymorphisms in carboxylesterase1A2 (CES1A2) gene involves specific protein 1 (Sp1) binding sites. Biochem Biophys Res Commun 369: 939–42. 10.1016/j.bbrc.2008.02.120 18328811

[19] Fukami T, Nakajima M, Maruichi T, Takahashi S, Takamiya M, Aoki Y, et al (2008) Structure and characterization of human carboxylesterase 1A1, 1A2, and 1A3 genes. Pharmacogenet Genomics 18: 911–20. 18794728 10.1097/FPC.0b013e32830b0c5e

[20] Sai K, Saito Y, Tatewaki N, Hosokawa M, Kaniwa N, Nishimaki-Mogami T, et al (2010) Association of carboxylesterase 1A genotypes with irinotecan pharmacokinetics in Japanese cancer patients. Br J Clin Pharmacol 70: 222–33. 10.1111/j.1365-2125.2010.03695.x 20653675 PMC2911552

[21] Laizure SC, Parker RB, Herring VL, Hu ZY. (2014) Identification of carboxylesterase-dependent dabigatran etexilate hydrolysis. Drug Metab Dispos 42: 201–6. 10.1124/dmd.113.054353 24212379 PMC3912543

[22] Zhu HJ, Appel DI, Peterson YK, Wang Z, Markowitz JS. (2010) Identification of selected therapeutic agents as inhibitors of carboxylesterase 1: potential sources of metabolic drug interactions. Toxicology 270: 59–65. 10.1016/j.tox.2010.01.009 20097249

[23] Zhu HJ, Wang X, Gawronski B, Brinda B, Angiolillo D, Markowitz J. (2013) Carboxylesterase 1 as a determinant of clopidogrel metabolism and activation. J Pharmacol Exp Ther 344: 665–72. 10.1124/jpet.112.201640 23275066

[24] Pare G, Eriksson N, Lehr T, Connolly S, Eikelboom J, Ezekowitz MD, et al (2013) Genetic determinants of dabigatran plasma levels and their relation to bleeding. Circulation 127: 1404–12. 10.1161/CIRCULATIONAHA.112.001233 23467860

[25] Bruxel EM, Salatino-Oliveira A, Genro JP, Zeni CP, Polanczyk GV, Chazan R, et al (2013) Association of a carboxylesterase 1 polymorphism with appetite reduction in children and adolescents with attention-deficit/hyperactivity disorder treated with methylphenidate. Pharmacogenomics J 13: 476–80. 10.1038/tpj.2012.25 22688218

[26] Juillerat L, Nussberger J, Menard J, Mooser V, Christen Y, Waeber B, et al (1990) Determinants of angiotensin II generation during converting enzyme inhibition. Hypertension 16: 564–72. 2172161 10.1161/01.hyp.16.5.564

[27] Biollaz J, Schelling JL, Jacot Des Combes B, Brunner DB, Desponds G, Brunner HR, et al (1982) Enalapril maleate and a lysine analogue (MK-521) in normal volunteers; relationship between plasma drug levels and the renin angiotensin system. Br J Clin Pharmacol 14: 363–8. 6289859 10.1111/j.1365-2125.1982.tb01992.x PMC1427615

[28] Delacretaz E, Nussberger J, Puchler K, Wood AJ, Robinson PR, Waeber B, et al (1994) Value of different clinical and biochemical correlates to assess angiotensin converting enzyme inhibition. J Cardiovasc Pharmacol 24: 479–85. 7528305 10.1097/00005344-199409000-00017

[29] Nussberger J, Brunner D, Keller I, Brunner HR. (1992) Measurement of converting enzyme activity by antibody-trapping of generated angiotensin II. Comparison with two other methods. Am J Hypertens 5: 393–8. 1326287 10.1093/ajh/5.6.393

[30] McMurray JJ, Adamopoulos S, Anker SD, Auricchio A, Bohm M, Dickstein K, et al (2012) ESC guidelines for the diagnosis and treatment of acute and chronic heart failure 2012: The Task Force for the Diagnosis and Treatment of Acute and Chronic Heart Failure 2012 of the European Society of Cardiology. Developed in collaboration with the Heart Failure Association (HFA) of the ESC. Eur J Heart Fail 14: 803–69. 10.1093/eurjhf/hfs105 22828712

[31] Plovsing RR, Wamberg C, Sandgaard NC, Simonsen JA, Holstein-Rathlou NH, Hoilund-Carlsen PF, et al (2003) Effects of truncated angiotensins in humans after double blockade of the renin system. Am J Physiol Regul Integr Comp Physiol 285: R981–91. 12869368 10.1152/ajpregu.00263.2003

[32] Sandgaard NC, Bie P. (1996) Natriuretic effect of non-pressor doses of endothelin-1 in conscious dogs. J Physiol 494 (Pt 3): 809–18. 8865076 10.1113/jphysiol.1996.sp021534 PMC1160679

[33] Yamada S, Richardson K, Tang M, Halaschek-Wiener J, Cook VJ, Fitzgerald JM, et al (2010) Genetic variation in carboxylesterase genes and susceptibility to isoniazid-induced hepatotoxicity. Pharmacogenomics J 10: 524–36. 10.1038/tpj.2010.5 20195289

[34] Barrett JC, Fry B, Maller J, Daly MJ. (2005) Haploview: analysis and visualization of LD and haplotype maps. Bioinformatics 21: 263–5. 15297300 10.1093/bioinformatics/bth457

[35] dbSNP Short Genetic Variations (NCBI). Available: http://www.ncbi.nlm.nih.gov/SNP.

[36] Wang X, Wang G, Shi J, Aa J, Comas R, Liang Y, et al (2015) CES1 genetic variation affects the activation of angiotensin-converting enzyme inhibitors. Pharmacogenomics J. 10.1038/tpj.2015.42 PMC6329299 26076923

[37] Tarkiainen EK, Tornio A, Holmberg MT, Launiainen T, Neuvonen PJ, Backman JT, et al (2015) Effect of carboxylesterase 1 c.428G > A single nucleotide variation on the pharmacokinetics of quinapril and enalapril. Br J Clin Pharmacol. 10.1111/bcp.12667 PMC4631185 25919042

[38] Geshi E, Kimura T, Yoshimura M, Suzuki H, Koba S, Sakai T, et al (2005) A single nucleotide polymorphism in the carboxylesterase gene is associated with the responsiveness to imidapril medication and the promoter activity. Hypertens Res 28: 719–25. 16419644 10.1291/hypres.28.719

[39] Ghosh S. (2000) Cholesteryl ester hydrolase in human monocyte/macrophage: cloning, sequencing, and expression of full-length cDNA. Physiol Genomics 2: 1–8. 11015575 10.1152/physiolgenomics.2000.2.1.1

[40] Blais DR, Lyn RK, Joyce MA, Rouleau Y, Steenbergen R, Barsby N, et al (2010) Activity-based protein profiling identifies a host enzyme, carboxylesterase 1, which is differentially active during hepatitis C virus replication. J Biol Chem 285: 25602–12. 10.1074/jbc.M110.135483 20530478 PMC2919124

[41] Crow JA, Middleton BL, Borazjani A, Hatfield MJ, Potter PM, Ross MK. (2008) Inhibition of carboxylesterase 1 is associated with cholesteryl ester retention in human THP-1 monocyte/macrophages. Biochim Biophys Acta 1781: 643–54. 10.1016/j.bbalip.2008.07.005 18762277 PMC2574903

[42] Okazaki H, Igarashi M, Nishi M, Tajima M, Sekiya M, Okazaki S, et al (2006) Identification of a novel member of the carboxylesterase family that hydrolyzes triacylglycerol: a potential role in adipocyte lipolysis. Diabetes 55: 2091–7. 16804080 10.2337/db05-0585

[43] Dolinsky VW, Gilham D, Alam M, Vance DE, Lehner R. (2004) Triacylglycerol hydrolase: role in intracellular lipid metabolism. Cell Mol Life Sci 61: 1633–51. 15224187 10.1007/s00018-004-3426-3 PMC11138677

[44] Bencharit S, Edwards CC, Morton CL, Howard-Williams EL, Kuhn P, Potter PM, et al (2006) Multisite promiscuity in the processing of endogenous substrates by human carboxylesterase 1. J Mol Biol 363: 201–14. 16962139 10.1016/j.jmb.2006.08.025 PMC1762004

[45] Bora PS, Guruge BL, Miller DD, Chaitman BR, Ruyle MS. (1996) Purification and characterization of human heart fatty acid ethyl ester synthase/carboxylesterase. J Mol Cell Cardiol 28: 2027–32. 8899561 10.1006/jmcc.1996.0195

[46] Law MR, Wald NJ, Rudnicka AR. (2003) Quantifying effect of statins on low density lipoprotein cholesterol, ischaemic heart disease, and stroke: systematic review and meta-analysis. BMJ 326: 1423 12829554 10.1136/bmj.326.7404.1423 PMC162260

[47] Hoshida S, Kato J, Nishino M, Egami Y, Takeda T, Kawabata M, et al (2001) Increased angiotensin-converting enzyme activity in coronary artery specimens from patients with acute coronary syndrome. Circulation 103: 630–3. 11156871 10.1161/01.cir.103.5.630

[48] Studer R, Reinecke H, Muller B, Holtz J, Just H, Drexler H. (1994) Increased angiotensin-I converting enzyme gene expression in the failing human heart. Quantification by competitive RNA polymerase chain reaction. J Clin Invest 94: 301–10. 8040271 10.1172/JCI117322 PMC296310

[49] Hirsch AT, Talsness CE, Schunkert H, Paul M, Dzau VJ. (1991) Tissue-specific activation of cardiac angiotensin converting enzyme in experimental heart failure. Circ Res 69: 475–82. 1650297 10.1161/01.res.69.2.475

[50] Zhuo JL, Li XC. (2011) New insights and perspectives on intrarenal renin-angiotensin system: focus on intracrine/intracellular angiotensin II. Peptides 32: 1551–65. 10.1016/j.peptides.2011.05.012 21699940 PMC3137727

[51] Sharman DC, Morris AD, Struthers AD. (2007) Gradual reactivation of vascular angiotensin I to angiotensin II conversion during chronic ACE inhibitor therapy in patients with diabetes mellitus. Diabetologia 50: 2061–6. 17676311 10.1007/s00125-007-0754-5

[52] Struthers AD. (1996) Aldosterone escape during angiotensin-converting enzyme inhibitor therapy in chronic heart failure. J Card Fail 2: 47–54. 8798105 10.1016/s1071-9164(96)80009-1

[53] Furberg CD, Pitt B. (2001) Are all angiotensin-converting enzyme inhibitors interchangeable? J Am Coll Cardiol 37: 1456–60. 11300461 10.1016/s0735-1097(01)01161-5

[54] Feldman RD, Hussain Y, Kuyper LM, McAlister FA, Padwal RS, Tobe SW. (2014) Intraclass Differences Among Antihypertensive Drugs. Annu Rev Pharmacol Toxicol. 10.1146/annurev-pharmtox-010814-124446 25251994

[55] Shah AD, Arora RR. (2005) Tissue angiotensin-converting enzyme inhibitors: are they more effective than serum angiotensin-converting enzyme inhibitors? Clin Cardiol 28: 551–5. 16405197 10.1002/clc.4960281203 PMC6654115

[56] Cressman AM, Macdonald EM, Fernandes KA, Gomes T, Paterson JM, Mamdani MM, et al (2015) A population-based study of the drug interaction between clopidogrel and angiotensin converting enzyme inhibitors. Br J Clin Pharmacol. 10.1111/bcp.12682 PMC4594702 25980448

[57] Williams GH, Cain JP, Dluhy RG, Underwood RH. (1972) Studies of the control of plasma aldosterone concentration in normal man. I. Response to posture, acute and chronic volume depletion, and sodium loading. J Clin Invest 51: 1731–42. 4338121 10.1172/JCI106974 PMC292320

[58] Waeber B, Nussberger J, Perret L, Santoni JP, Brunner HR. (1989) Experience with perindopril in normal volunteers. Arch Mal Coeur Vaiss 82 Spec No 1: 35–41. 2549899

[59] Laizure SC, Herring V, Hu Z, Witbrodt K, Parker RB. (2013) The role of human carboxylesterases in drug metabolism: have we overlooked their importance? Pharmacotherapy 33: 210–22. 10.1002/phar.1194 23386599 PMC4572478

[60] Takahashi S, Katoh M, Saitoh T, Nakajima M, Yokoi T. (2009) Different inhibitory effects in rat and human carboxylesterases. Drug Metab Dispos 37: 956–61. 10.1124/dmd.108.024331 19225040

[61] Wang X, Zhu HJ, Markowitz JS. (2015) Carboxylesterase 1-mediated drug-drug interactions between clopidogrel and simvastatin. Biol Pharm Bull 38: 292–7. 10.1248/bpb.b14-00679 25747989

[62] Singh BN, Malhotra BK. (2004) Effects of food on the clinical pharmacokinetics of anticancer agents: underlying mechanisms and implications for oral chemotherapy. Clin Pharmacokinet 43: 1127–56. 15568891 10.2165/00003088-200443150-00005

[63] Ogawa R, Stachnik JM, Echizen H. (2014) Clinical pharmacokinetics of drugs in patients with heart failure: an update (part 2, drugs administered orally). Clin Pharmacokinet 53: 1083–114. 10.1007/s40262-014-0189-3 25248847

